# St. Mary’s Hospital Medical School

**Published:** 1861-09

**Authors:** 


ST. MARY'S HOSPITAL MEDICAL SCHOOL.—A kind friend in London has sent us the annual announcement of this school for 1861–2, from which we learn that it is in a highly flourishing condition, as might, indeed, be supposed it would be, when we look at the imposing array of names composing its faculty and the advantages afforded by St. Mary's Hospital as a field for clinical instruction. Many of the teachers are writers of eminence, well known to the profession of this country. The branches of study are clinical medicine and surgery, medicine and surgery, physiology, anatomy, operations on the dead body, dissections, chemistry, materia medica, botany, midwifery, ophthalmic surgery, aural surgery, dental surgery, medical jurisprudence, comparative anatomy, and natural philosophy. The faculty consists of Messrs. Alderson, Chambers, Sibson, Coulson, Lane, Ure, Gascoyen, James Lane, Spencer Smith, Broadbent, Haynes Walton, Field, Sircombe, Norton, Sieveking, Hewitt, Tyler Smith, Dresser, Sanderson, Smalley, White, Cooper, and Toynbee.

The school has two sessions, a winter and a summer. The former commences on the 1st of October, and terminates on the 31st of March; the latter on the 1st of May and the 31st of July. The post-mortem examinations are performed by the curator of the museum, the number of bodies annually inspected being nearly 200. Clinical teaching occupies a prominent rank in the curriculum of studies.

